# Days, Schedules, and Attention: How Time Affects Mobile News Consumption Among Young Swiss People

**DOI:** 10.1177/20501579251376418

**Published:** 2025-09-25

**Authors:** Morley Weston, Daniel Vogler, Adrian Rauchfleisch, Pascal Jürgens, Mark Eisenegger

**Affiliations:** 1 Department of Communication and Media Research (IKMZ), 27217 University of Zurich, Zurich, Switzerland; 2 Research Center for the Public Sphere and Society (fög), 27217 University of Zurich, Zurich, Switzerland; 3 Graduate Institute of Journalism, National Taiwan University, Taipei, Taiwan 33561 (ROC); 4 Department of Media Research, University of Trier, Trier, Germany 26595

**Keywords:** Mobile tracking, news use, social media, Bayesian analysis, computational methods

## Abstract

News consumption is often treated as static behavior, yet situational and temporal factors play a significant role in shaping how individuals consume news throughout their daily lives. Mobile phones have become a driving force of dynamic news habits, especially among younger audiences. This study aims to bridge the gap between static perceptions of news consumption and the dynamic realities of daily mobile usage. To investigate this, we tracked news usage on the mobile phones of 384 young adults in Switzerland over three weeks in 2021, in a period surrounding a national referendum. These data were combined with two surveys at the beginning and end of the tracking period to examine how a political referendum, daily schedules, and browsing habits are related to the amount of mobile news consumption. Using a Bayesian time-lagged model, we found an increase in news consumption on the day of the referendum, and a slight decrease on weekends and during working hours. We also found that participants read more news when they first opened their phones than as a browsing session continued, and that higher news consumption was substantially associated with the use of X/Twitter and Instagram, but only slightly with TikTok.

## Introduction

The news that individuals consume is often studied as a static quantity, but the rhythms of life play an important role in news consumption (Peters, 2012). Waves of increased news consumption appear and abate following crises and wars (Althaus, 2002; Nelson & Lewis, 2022) or in the run-up to important political events like elections (Haugsgjerd & Karlsen, 2024). Additionally, individual daily routines like weekends vs. weekdays (Weaver et al., 2019), working hours (Boczkowski, 2010; Zhang & Ha, 2016), and leisure time (Boczkowski et al., 2018) are related to news consumption. The situational differences in how and why individuals consume news have been changed greatly by the growing prevalence of smartphones and their ubiquity in day-to-day life. Especially for the younger generation, mobile devices have become the main way to access news (Klopfenstein Frei et al., 2022; Schwaiger et al., 2022).

Smartphones have facilitated the possibility of consuming news from an abundance of different sources at any time and any place. Substantial numbers of users indicate that they use their smartphones to access news on public transport, in bed, and even on the toilet (Newman et al., 2017). Therefore, mobile news usage may be particularly affected by certain national events (Ohme, 2020), related to certain times of the day (Boczkowski et al., 2018; Zhang & Ha, 2016), and show distinct patterns even within sessions (Ohme et al., 2016). Dynamic mobile news usage patterns are often studied with qualitative methods, such as interviews or diaries (e.g., Boczkowski, 2010). Comprehensive quantitative studies are few and far between, also due to methodological challenges. Studies with behavioral data, therefore, often focus on patterns within sessions that appear in the data; for instance, incidental news exposure following the use of social media (Merten et al., 2022). Few studies have yet combined tracking data with surveys to measure mobile news consumption precisely and at the same time gather information about the context of usage. Overall, the existing research into how daily schedules or external events affect (mobile) news consumption remains scarce (Groot Kormelink & Costera Meijer, 2020).

This study seeks to address these gaps by investigating the temporal dynamics of mobile news consumption of young adults. Our sample group comprised people aged 18 to 25 living in Switzerland. This younger cohort is of particular interest because of their heavy reliance on the smartphone for consuming news. Specifically, we explore: (1) how mobile news usage responds to national events, (2) variations in news consumption by time of day and (3) browsing session, as well as (4) interactions between news consumption and social media use. To accomplish this, we used surveys and tracking data to deduce participants’ rhythms of mobile phone use and combined this with news browsing data. We relied on a custom method to track the mobile news consumption, both in apps and on internet browsers, of 384 participants. We then examined what periods in time they were most likely to visit a news website, using a Bayesian multilevel model.

## Literature Review

Digitization and the rise of platforms, search engines, and messenger apps have fundamentally changed the way people consume news (Goyanes, 2020; Kleis Nielsen & Ganter, 2018; Lee et al., 2014; Merten et al., 2022; Ohme & Mothes, 2023). These developments have created high-choice media environments (Aelst et al., 2017), offering abundant content and enabling individuals to curate their personal news diets (Schrøder, 2015). The ubiquitous access enabled by mobile phones has not only amplified these individualized patterns but also embedded news consumption seamlessly into daily routines (Boczkowski et al., 2018; Zhang & Ha, 2016).

### Dynamic Mobile News Consumption

This study looks at news use as a dynamic phenomenon, specifically targeting differences between days, time of day, and within a mobile phone usage session. We focus on young adults as our primary subject group. This demographic has attracted significant attention among researchers due to their strong reliance on the smartphone and social media, low news use, and incidental news consumption (e.g., Fletcher & Nielsen, 2017; Haugsgjerd & Karlsen, 2024). Young adults are also of both scholarly and practical interest as they represent the future audience of news media. Understanding the consumption patterns of this generation is therefore particularly relevant. In the following sections, we provide an overview of the literature on temporal patterns of (mobile) news in general and ask whether these patterns also apply to young adults.

### Influence of Political Events

Addressing dynamic news consumption as a phenomenon, we first look at differences between days. More specifically, we investigate whether news consumption on the day of an important and planned political event is higher than on ordinary days. Haugsgjerd and Karlsen (2024), for instance, showed a growth in news consumption among Norwegians in the run-up to the 2017 national elections. Although the study confirmed a general age gap – meaning younger individuals consumed less news than older ones – it also found that this gap narrowed as the election approached. This suggests that younger adults’ news consumption is more responsive to political events. Although a longer-term event, multiple studies saw a significant increase in news use during the early days of the COVID-19 pandemic (Broersma & Swart, 2022; Van Aelst et al., 2021; Ytre-Arne & Moe, 2021), implying that major events are associated with individuals turning to the news. Regarding age, Van Aelst et al.’s (2021) study showed that older individuals were more likely to increase their news consumption during the pandemic than younger cohorts.

Switzerland is a compelling case for studying the influence of political events on news consumption, as referendums on the national level take place several times per year. Reading the news is still among the most common ways for citizens to inform themselves about such referendums (gfs.Bern, 2021). Therefore, Swiss news outlets invest considerable resources on dedicated coverage in the run-up to referendum. We thus investigate whether such special phases in the political process reveal different patterns in how much news people consume. As is often the case, several referendums are held on the same day. In our case, Swiss voters had to decide on raising taxes for companies and the legalization of same-sex marriages, a policy that was especially popular among younger audiences. We therefore propose the following question:

Research question 1: On the day of the national referendum, are young adults more likely to consume news on their smartphones?

### Influence of Daily Schedules

The effect of time of day on news consumption has been studied since online news was in its nascence. News editors are shown to pay close attention to audience metrics and focus on publishing certain stories at different times of day to maximize readership (Thurman & Myllylahti, 2009). These “rhythms of news production” (Fürst, 2020, p. 272) have become ubiquitous in the newsroom, and timing stories an important part of editorial strategy (Belair-Gagnon, 2019).

Some studies suggest that audiences may structure their news consumption around specific times of day. In an early study that included mobile phone usage, Dimmick et al. (2011) found that news over “multimedia mobile” devices was more likely to be consumed during daytime hours, as well as on public transport. In interview-based studies, Van Damme et al. (2015) and Ytre-Arne (2023) both showed that some participants routinely checked several online newspapers as part of their morning routine. Schnauber-Stockmann and Mangold (2020) highlight that news use on a smartphone was likely to be a part of a daily routine, regardless of whether one was alone or not, or whether one was busy with other obligations.

Online news consumption was observed to be interwoven throughout the day, rather than a separate activity (Diddi & LaRose, 2006). So-called “news snacking,” frequent and shallow patterns of news consumption, is shown to be typical on mobile devices (Molyneux, 2018; Ohme & Mothes, 2023). Peng and Zhu (2020) examined mobile phone usage as a sequential process and found that, over the course of a day, participants were more likely to reengage with their phones, defined as closing and reopening their phones within a short period of time, in the middle of the day.

However, daily routines vary between individuals, with age being possibly one of the main explanatory factors. The schedules of young adults usually look very different from those of older cohorts as they still go to school or university or have just entered working life, and pursue different leisure activities. First, we ask if weekdays differ from weekends, as daily routines follow different patterns and are shown to affect media consumption, for example, general social media use (You et al., 2023) and smartphone usage of young people (Külling-Knecht et al., 2024).

Research question 2: Do young adults read more news on their smartphones on weekdays compared to weekends?

News consumption patterns also differ within days (Boczkowski, 2010; Peters, 2012; Zhang & Ha, 2016). For instance, news can be consumed on mobile devices during the commute and maybe (not) at work or during school. We can easily think of peculiarities of the daily schedules of young adults; for instance, working in the evening, more leisure time, and restrictions by parents, which might influence news consumption on the smartphone. However, empirical evidence from quantitative studies on this topic for young adults is scarce. We therefore ask the following explorative research questions:

Research question 3: Do young adults read more news on their smartphone at some times of the day compared to others?

Research question 4: Do young adults read news for longer periods at some times of the day compared to others?

Finally, we ask if working hours are related to the prevalence of news consumption. One of the few studies that addresses this issue, by Boczkowski (2010), shows that some people read news exclusively during work and some not at all. Differences are related to the type of work, restrictions by employers, or personal obligation, but also job-relevant news consumption (Loi et al., 2024). The availability of smartphones presumably changes these patterns as using personal devices for news consumption might be felt as more opportune in a work setting. However, up to now, studies have not explicitly looked at young adults who have different working routines, and neglected the role of the smartphone. Therefore, we formulate an open research question:

Research question 5: Do young adults read more news on their smartphone at work compared to other daytime hours?

### Within-session Influences

Previous studies have differentiated between intentional and unintentional mobile phone use, and we can use participants’ behavior to measure this. In their sequential study, Peng and Zhu (2020) also found that users were likely to begin a browsing session with an app for a specific purpose, and then gravitate toward social media, games, or other types of app. They found that most people opened their phones for communication and tools but often continued with social media apps. This phenomenon was also examined by Terzimehic et al. (2023), calling it the “mobile phone rabbit hole,” led on by dopamine-driven feedback loops and a need for short-term gratification. They found that 25.8% of mobile phone usage sessions were “rabbit hole sessions” – those longer than intended, with a greater chance of reported loss of time, space, and agency. Almoallim and Sas (2023) differentiate between meaningful smartphone use, related to “connectedness, coordination, and functional or pragmatic purposes such as work, productivity or getting things done, information needs, and learning and self-development” (p. 255) and meaningless smartphone use as a result of stress or boredom. News use can fall into either category; it certainly is an information need but can also be a result of boredom.

There are competing models of hypotheses related to mobile news usage. People could visit news websites as an intentional act, and users would be more likely to visit news websites at the beginning of a browsing session. Alternately, news use could be largely incidental, and users’ visits to news websites would be dispersed throughout a phone usage session. Research shows that incidental news exposure has risen in recent years, especially among young people, with social media as a main driver (Fletcher & Nielsen, 2017). Boczkowski et al. (2018) found that it was common for young adults to come across news incidentally on social media, and that incidental news use was associated with “fragmentary reading patterns, loss of hierarchy of the news, and coexistence of editorial, algorithmic, and social filtering” (p. 3523). However, another study by Mitchelstein et al. (2020), found a continuum of incidental news exposure; for example, participants were more likely to be exposed to news if they had a digital environment that allowed for more exposure, such as following news websites on social media or accepting notifications.

Quantitative studies back up the importance of incidental news exposure on social media to news consumption. A tracking study of desktop web browser behavior (Merten et al., 2022) found that, when browsing social media, 4% of newsfeed items were related to a news outlet, and that this was likewise correlated with political interest. As a large portion of news consumption of young adults and adolescents happens through social media (Klopfenstein Frei et al., 2022; Schwaiger et al., 2022), we formulate the following hypotheses:

Hypothesis 1: Participants will read more news at the beginning of a browsing session than at the end.

Hypothesis 2: Time periods of social media usage will be associated with a greater propensity to read news.

## Methods

To address our research questions and test our hypotheses, we developed a method to track mobile phone usage by recording all network connections between participants’ phones and web servers. Participants used their phones while connected to a virtual private network (VPN) managed by our research team. This setup anonymously logged all domains visited, whether through apps or web browsers, which is especially important given that young people have been found to predominantly use apps over their web browsers for checking news (Fichte & York, 2024). This approach offered several advantages; it captured a comprehensive view of mobile phone activity across platforms while maintaining participant anonymity and privacy. The tracking data were paired with surveys before and after the study, administered online to preserve anonymity.

Our subject of study is young people in Switzerland aged 18 to 25. Participants were recruited primarily via Instagram advertisements in French and German. Of the respondents, 75.5% lived in German-speaking Switzerland, whereas 24.5% lived in the French-speaking region, which represents valid quota for the two regions (73% vs. 27%). Due to the methods of recruitment through Instagram, we over-sampled for women in our study, who formed 63% of our sample. The average age was 21.1 years. Students were overrepresented in the panel at 69.3%. 12.0% of the participants were completing an apprenticeship or had permanent jobs, and 8.3% were pupils. Despite some skew, the sample represented a significant amount of variation across sociodemographic and regional variables and should support robust regression analyses, especially with controls. The dropout rate was quite high, and of the 771 participants who signed up, only 384 successfully sent sufficient data (at least five days of tracking data) to be included in the study. Participants were given CHF 50 (approximately USD 50) in compensation upon completion of tracking and two surveys. Approval for this project was granted by the Ethics Committee of the Faculty of Arts and Social Sciences of the University of Zurich. Tracking occurred over three weeks, from September 14 to October 4, 2021.

### Data Processing

The data collected were in the form of individual web requests between mobile phones and all websites or apps visited over the course of this study. This is a highly uneven unit of analysis, as some dynamic websites like Facebook or Instagram process dozens of requests per minute from each device, while some static websites only process a web request when a user actively clicks on a link. In our data, for example, during a minute in which a participant visited Instagram, they would send a mean of 2.679 requests to the website, with a minimum of 1 and a maximum of 57. Similar numbers were found for TikTok (mean: 4.666, max: 45) and slightly fewer for X/Twitter (mean: 2.783, max: 19). However, news websites showed more variation, with national broadcaster SRF (mean: 2.116, max: 68) having a much higher maximum than Zurich-based newspaper NZZ (mean: 3.001, max: 11). The minimum for all websites described here was 1. Thus, comparing raw requests in this context would be meaningless; more dynamic websites would be weighted much more heavily than more static, text-based ones. To normalize these unbalanced web data, we divided mobile phone usage into time blocks of increasing specificity: days, time of day, individual browsing sessions, and ultimately 10-minute segments, which became the final unit of analysis.

### Operationalizing Mobile Phone News Use

To investigate the dynamics of mobile news consumption, we transformed the raw tracking data into a set of operationalized variables that represent the key mobile use and combined it with survey data on demographic factors and working hours. Below, we detail how each variable was constructed and its role in statistical modeling. A summary of all variables is provided in Table 1.

**Table 1.** Summary Statistics for All Variables Used in Modeling.

Variable	Min	Mean	Max	SD	Description
Age at survey	18.00	21.11	25.00	2.11	Age of the user, gathered from survey data.
Session progress	0.00	0.52	1.00	0.31	Progress within the session, as a ratio of 0 (participant begins using phone) to 1 (participant stops using phone).
Time online ratio	0.52	0.92	1.00	0.08	Ratio of time online within 10-min block. Zero represents no time online, 1 represents all time online.
Gender: (women: 63%, men: 37%)					Gender, gathered from survey data. Summarized at user level.
Read news: (no: 95%, yes: 5%)					Whether the user read news during the 10-min block.
Read news (lagged): (no: 95%, yes: 5%)					Whether the user read news during the previous 10-min block.
Is weekend: (weekday: 74%, weekend: 26%)					Whether the session occurred on a weekend.
Is referendum day: (no: 96%, yes: 4%)					Whether the session occurred on the day of the referendum
Time block: (Before 9:00: 5.632%, 09:00 to 11:59: 17.698%, 12:00 to 14:59: 19.338%, 15:00 to 17:59: 19.804%, 18:00 to 20:59: 19.390%, 21:00 and after: 18.138%)					Time of day, per participant's schedule.
At work: (no: 90%, yes: 10%)					Whether the user was at work during the session, estimated from survey data.
Visited X/Twitter: (no: 95%, yes: 5%)					Whether the user visited X/Twitter during the 10-min block.
Visited Instagram: (no: 72%, yes: 28%)					Whether the user visited Instagram during the 10-min block.
Visited TikTok: (no: 93%, yes: 7%)					Whether the user visited TikTok during the 10-min block.

Data were complete for all users (*n* = 384).

#### News Use

Prior to starting the study, we assembled a list of 3,778 news websites from several sources; namely the Swiss Media Quality Yearbook (Jahrbuch Qualität der Medien, 2023), ABYZ News Links (www.abyznewslinks.com), and The Media Cloud Project (Roberts et al., 2021). When paired with user browsing data, users were found to have visited 497 distinct news sites over the course of the study. News use was recorded as a binary variable, indicating whether a participant visited at least one news website within a 10-minute period. Importantly, this binary measure captures the occurrence of news use during a segment, not the duration or intensity of engagement. In total, 27,417 of 506,033 10-minute blocks (5.42%), included some news use, and this was distributed unequally among participants. For the average participant, 5.16% of time blocks online included some news use, with a standard deviation of 6.88%. The minimum amount of time reading news was 0 (five participants read no news), and the maximum was a participant for whom 43.00% of their 10-minute blocks included some news use. Just 51 of the 384 participants accounted for half the time spent reading news in total, and the Gini coefficient for total 10-minute blocks among users was 0.606.

#### Lagged News Use

To account for the sequential nature of the data, we included a lagged news use variable, which indicates whether a participant accessed a news website in the preceding 10-minute block. Including this variable controls for the autocorrelative tendency of mobile phone use, as individuals who read news in one time block are likely to continue doing so in subsequent blocks. By capturing this temporal dependency, the model's robustness is improved, ensuring that patterns in news consumption are not overstated in our model.

#### Social Media Use

Social media use was operationalized by identifying visits to Instagram, TikTok, and X/Twitter, the three social media platforms that young people in Switzerland use most often to access news (Jahrbuch Qualität der Medien, 2023). For each 10-minute block, we recorded whether a participant accessed one or more of these platforms, coding each as a separate dummy variable. This approach allowed us to capture overlaps in platform usage within the same time segment and assess their potential interactions with news use.

#### Total Time Online

We included the total time spent online within each 10-minute segment as a variable in the model. This was scaled from 0 (no time spent online) to 1 (10 out of 10 minutes in the segment were spent on the phone). This adjustment accounts for the influence of overall online activity on the likelihood of news consumption, as individuals are naturally more likely to access a news article when they spend more time online.

### Control Variables

Because the focus of this study is specifically on the situational factors affecting news consumption, we kept the demographic variables at the user level to a minimum. Previous studies have established that age (Boulianne & Shehata, 2022; Edgerly, 2017) and gender (Ohme & Mothes, 2023; Toff & Palmer, 2019) are correlated with news consumption, and we included these as control variables in this model as they explain a fair amount of the variation in news use and help with model convergence and accuracy.

### Dividing Mobile Usage Into Time Segments

Modeling daily fluctuations in mobile phone usage required splitting mobile phone activity and news consumption into periods of days, hours, and individual browsing sessions on devices. To capture these dynamics, we divided the dataset into multiple temporal segments, each designed to reflect distinct aspects of mobile behavior. These time-based measures allow for detailed analysis of how factors such as proximity to a national event, daily routines, and individual browsing sessions influence news consumption.

#### The Day of A National Event

To measure whether news usage changes in response to a national event, we added a binary variable to whether a session was on the day of the referendum.

#### Time of the Day

To check the effects of the time of day on mobile use, we divided these segments into three-hour blocks depending on time of day, from 6:00 to 8:59, 9:00 to 11:59, 12:00 to 14:59, 15:00 to 17:59, 18:00 to 20:59, and 21:00 to midnight, following the methodology of Van Damme et al. (2015) and Dimmick et al. (2011). These were coded as categorical variables, using the time block starting at 12:00 as a baseline measurement. Mobile phones continue to transmit a small amount of passive data (checking for messages, sending notifications, etc.) while participants were presumably sleeping, and these data created unreliable estimates of mobile phone use, where they would normally be drowned out by legitimate phone use. To counter these unreliable data, we excluded data from midnight to 6:00, when there was typically a decrease in mobile phone activity.

#### Time At Work

In surveys, we asked whether a participant was employed on a regular schedule, and if so, what hours they typically worked. We used this to code time blocks into working and non-working hours, as a binary variable.

#### Individual Browsing Sessions

**Figure 1.**
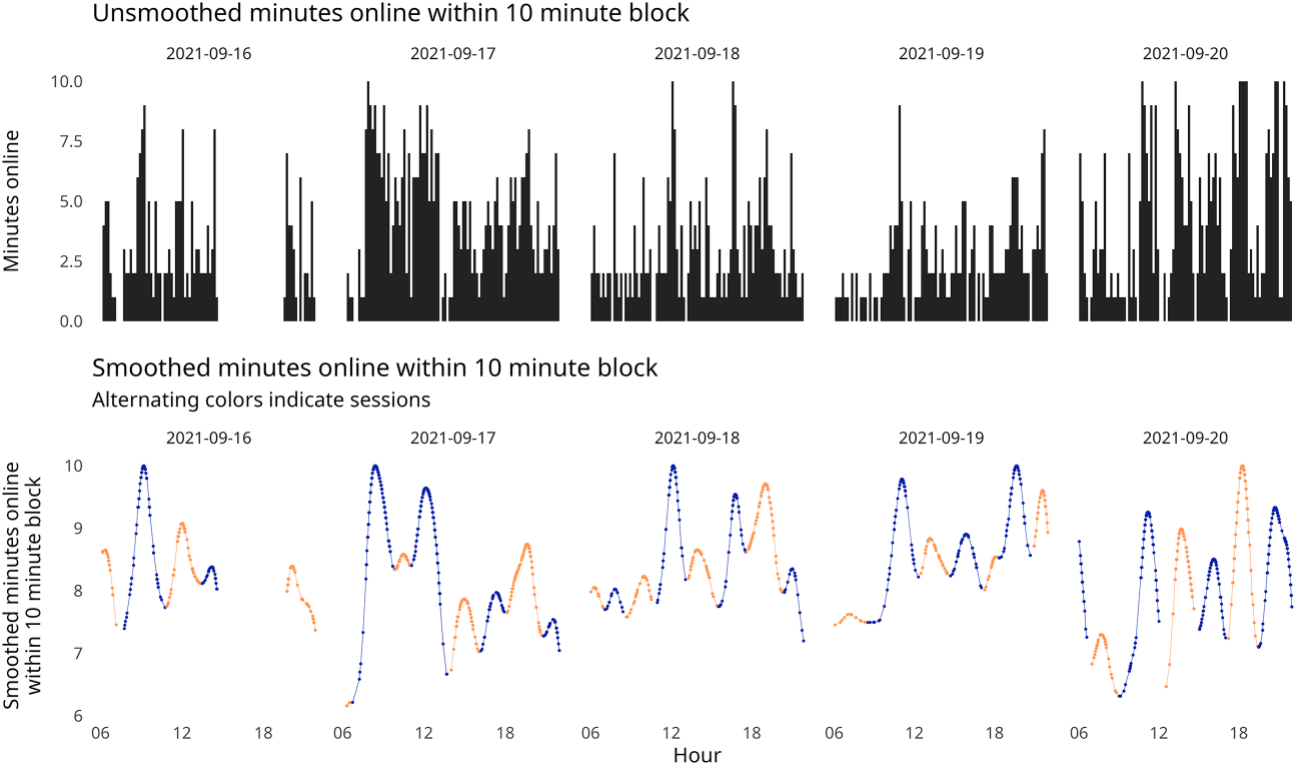
Session Detection from Mobile Use Data.

Note: Gaussian smoothing and peak detection was used to separate internet use into distinct sessions. First, the number of minutes online per 10-minute segment was calculated (above). Then, this was smoothed over, and valley detection on the smoothed data was used to determine individual sessions (below). A 5-day period for one user is shown for illustration.

As an even more granular measure of time-based internet use within our study, we split smartphone use into individual browsing sessions: periods between which a participant would pick their phone up and use it and when they would put it down. To do this, we first gathered the minutes of active network use and totaled these into 10-minute segments, giving a ratio of minutes per 10 minutes online. To divide these into browsing sessions, we then applied Gaussian smoothing to this data and then used peak and valley detection to create cut points of low news use. Smoothing was a necessary component of this because the raw ratios of news use were quite noisy and would have resulted in many short sessions. For an illustration of this process, see Figure 1.

We then divided this into sessions based on the local minimum of each peak and valley. Another tracking study with similar implementation (Peng & Zhu, 2020) conceptualized sessions differently from this study; when a user closes then immediately reopens their phone, which they term “mobile reengagement,” they consider this as a separate session. However, as our study relies on network requests to judge usage, we conceptualize browsing sessions as longer periods of continued phone use.

#### Ten-minute Segments

Within each of these sessions, we split mobile usage into 10-minute intervals as our final unit of measure. As the analysis of these data is largely based on time-based measures, this gives us enough fidelity to estimate at what time someone viewed a news article, while greatly reducing the size and noisiness of the dataset, reducing it from 13 million observations to 622,824. To test whether the model was sensitive to the segments being specifically 10 minutes, we ran a sensitivity analysis, detailed in Appendix 3 and illustrated in Figure A4.

#### Session Progress

We also divided each 10-minute block into browsing sessions: periods between when a participant picked up a phone and when they stopped using it. This was estimated entirely through their mobile phone use: minute-by-minute news use was first totaled per participant, then a Gaussian smoothing filter was applied to the total minutes online. Breaks were then cut in each local minimum in news use, so that time periods throughout the day were divided into separate sessions of news use. Because these browsing sessions were of different lengths, we coded this as the beginning of a browsing session being 0, and the end being 1, with the beginning of each 10-minute segment being some number in the middle. For example, a segment with a value of 0.1 would start at minute 12 of a 2-hour session.

### Modeling

For this analysis, a Bayesian modeling approach was taken, allowing for easier interpretation of results within our multilevel framework, easier convergence, and richer, more informative results. To establish a baseline for modeling the likelihood of a participant opening a news website within a given 10-minute segment, we first fitted a Bayesian model with three foundational variables: session progress, total time online, and lagged news consumption. This and all subsequent models were fitted using the brms package in *R*. Posterior distributions can be seen in Table A2.

As the outcome variable was binary, we used a logit-linked Bernoulli distribution as our likelihood function. Priors for all variables were set as Cauchy distributions centered (χ) on 0 with a scale parameter (λ) of 2.5, following Gelman et al. (2008). The modeling was done using the brms (Bürkner, 2017) package in *R*. Convergence was measured using the Gelman–Rubin statistic (*R*-hat) (Gelman & Rubin, 1992), with all successfully converging below 1.01.

From this basic model, we can draw a few key conclusions. Participants were more likely to visit a news website at the beginning of a browsing session than at the end. Furthermore, reading news in the previous 10-minute block was a strong predictor of reading news in the current block, highlighting the autocorrelative nature of news consumption. Lastly, spending more time online was positively correlated with increased news consumption.

#### Full Model

**Figure 2.**
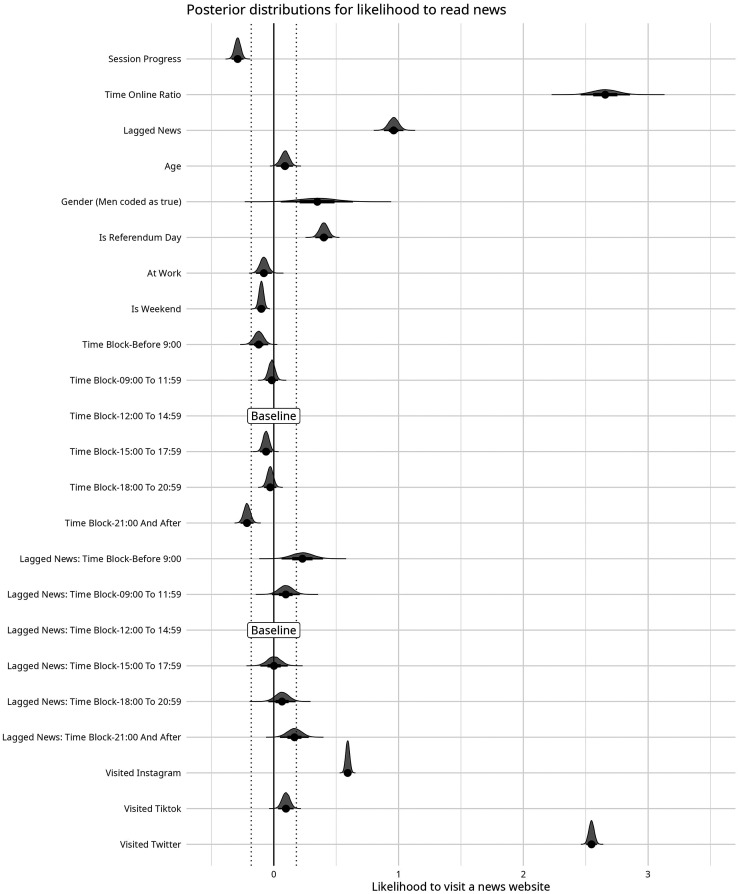
Posterior Distributions for the Final Model.

Note: Distributions that do not cross the 0 line can be considered credible. The region of practical equivalence (ROPE) is marked with dotted lines. Within this region, effects can be considered credible, but too small to be of practical interest. As categorical variables, the time block from 12:00 to 15:00 was chosen to be absorbed into the intercept.

**Figure 3.**
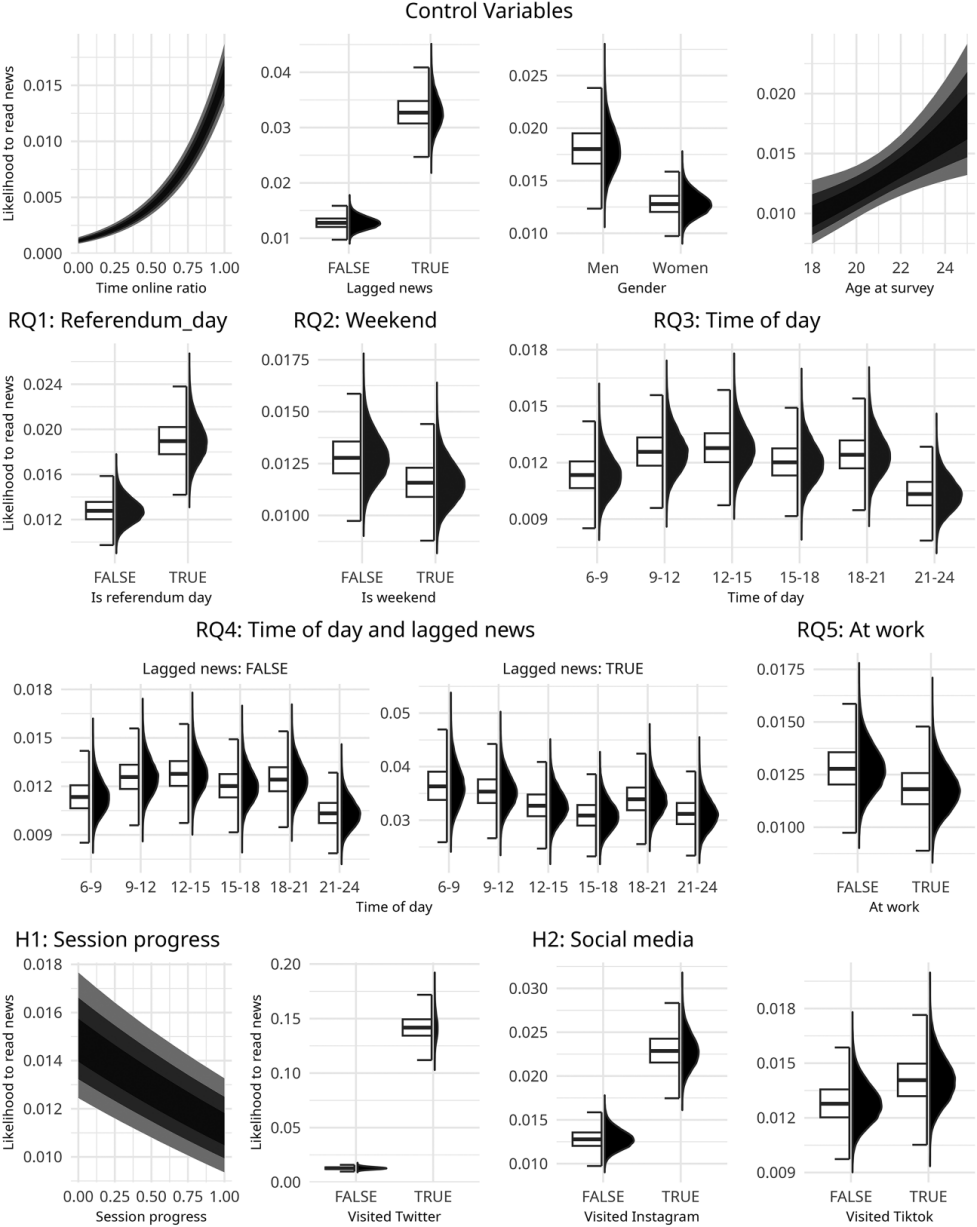
Conditional or Marginal Effects for Each variable.

Note: In this diagram, marginal effects of each variable are calculated separately for each independent variable. For categorical variables, the most common category was used. In this way, they represent the effect of each variable on a typical participant.

Building on this, we then expanded the model to include variables to address our research questions and hypotheses, namely age, gender, whether a news visit occurred on the day of the referendum, time of day, whether someone was at work or not, whether a news visit occurred on a weekend day, and which social media websites had been visited. To test for continuous news consumption by time of day, an interaction effect was included between the lagged news variable and time of day. Priors, as before, were set as Cauchy distributions centered on 0 with a scale parameter of 2.5. This choice reflects an assumption that time-related variables, contextual factors, and demographic characteristics may have weak or negligible effects on news consumption. Centering the variables on 0 facilitated the testing of credible and practically meaningful effects. Because of the increased number of parameters, the model was run for 2,000 warm-up iterations and 10,000 total iterations. All *R*-hats were below 1.01. Model specifications are shown in Appendix 2, and posterior distributions for the full model can be seen in Figure 2 and Table A3 (see Appendix). Marginal and conditional effects, and the simulated effect of each on an average participant, can be seen in Figure 3.

## Results

The full model allows us to answer our initial research questions. First, we asked if on days of the national referendum individuals were more likely to consume news (**RQ1**). We found a substantial increase in news use on the day of the referendum compared to the typical day (*β* = 0.40, 95% CI [0.33, 0.47]).

We then asked if participants read more news on weekdays compared to weekends (**RQ2**). We found that participants were less likely to read news on weekends compared to weekdays (*β* = −0.10, 95% CI [−0.14, −0.064]), but that this result fell entirely in the region of practical equivalence (ROPE) and was too small to be considered reliable.

Next, we turned to **RQ3**, whether participants read more news at some times of the day compared to others. Dividing the data into three-hour blocks by time of day, we found that participants consumed less news from 6:00 to 9:00 (*β* = −0.12, 95% CI [−0.20, −0.046]). However, they consumed about the same from 9:00 to 12:00 (*β* = −0.016, 95% CI [−0.065, 0.033]) as our baseline measurement of 12:00 to 15:00. From 15:00 to 18:00, they consumed less than the baseline measurement (*β* = −0.062, 95% CI [−0.11, −0.015]), about the same as baseline from 18:00 to 21:00 (*β* = −0.028, 95% CI [−0.076, 0.02]), and somewhat less afterward (*β* = −0.21, 95% CI [−0.27, −0.16]). However, these results largely fell within the region of practical equivalence and were not substantial. As explained in the methods section, data from midnight to 6:00 were excluded from our measurements as they were found to be unreliable.

We then examined whether participants read news for longer periods at some times of the day compared to others **(RQ4)**. Using the interaction effect between lagged news (whether someone had read news in the previous 10-minute block) and the time of day, we saw that participants were less likely to read news for a consecutive period of time before 9:00 (*β* = 0.23, 95% CI [0.063, 0.39]), but more likely from 9:00 to 12:00 (*β* = 0.096 95% CI [−0.014, 0.21]). With 12:00 to 15:00 as our baseline measurement, they consumed about the same amount consecutively from 15:00 to 18:00 (=0.0019, 95% CI [−0.11, 0.11]), and progressively more from 18:00 to 21:00 (*β* = 0.066, 95% CI [−0.042, 0.17]) and from 21:00 to midnight. (*β* = 0.17, 95% CI [0.05, 0.28]) This measure of consecutive reading differs from the total amount read, where the most was read during the mid-morning hours. As with the total amount read, these results fell largely within the region of practical equivalence and were not substantial.

Next, we examined whether the participants read more news at work compared to other daytime hours **(RQ5)**. We found that individuals were slightly less likely to read news during working hours (*β* = −0.08, 95% CI [−0.14, −0.017]). Similarly to the time of day, this effect was too small to be considered credible.

Next, we addressed whether participants read more news at the beginning of a browsing session than at the end **(H1)**. We found a substantial negative relationship between the progress of a browsing session and likelihood of consuming news (*β* = −0.29, 95% CI [−0.34, −0.24]). As a person continued to browse throughout a browsing session, they were less likely to visit a news website.

Finally, we examined whether time periods of social media usage were positively associated with greater propensity to read news **(H2)**. Visiting X/Twitter (*β* = 2.55, 95% CI [2.50, 2.59]) and Instagram (*β* = 0.59, 95% CI [0.56, 0.62]) in the previous 10 minutes were all associated with a substantially greater likelihood to visit a news website. However, the use of TikTok (*β* = 0.097, 95% CI [0.036, 0.16]) only showed a weak positive relationship with news consumption and fell within the region of practical equivalence. Thus, H2 is only supported for X/Twitter and Instagram.

## Discussion

This study identified several time-centered factors influencing the propensity to visit news websites or apps. First, the day of the national referendum was shown to be associated with a substantial increase in news consumption, which reflects findings from earlier studies on the relation between news consumption and planned political events (Haugsgjerd & Karlsen, 2024; Weaver et al., 2019). Further, time of day had a small effect, but not one that could be considered substantial. While we found that attention was held longer on the news during some times of the day over others, the results were too small to be considered consequential. This result, however, was largely in line with findings by Dimmick et al. (2011). The weak effect implies that news use has become routinized on mobile phones, with intermittent news use and news snacking for mobile news consumption (Ohme & Mothes, 2023). This finding contrasts with the findings of several interview studies (Van Damme et al., 2015; Ytre-Arne, 2023), which found that many people consume news in the morning as part of a daily routine. Evidence of routinized effects was also echoed in the finding that within individual browsing sessions – between when someone picked up and put down their phone – people read more news at the beginning than at the end. Finally, mobile use on the weekend and while at work had perceptible, but insignificant, effects. There are two possible reasons that participants’ work schedules had a weak effect on their news usage. First, the data were collected during 2021, when many people in Switzerland were still working from home or otherwise had their schedules affected by the COVID-19 pandemic. Second, this occurred during a travel holiday for many in the country.

Browsing session dynamics also revealed important insights. Participants were more likely to visit news websites at the start of a session than later, supporting the “mobile phone rabbit hole” phenomenon described by Terzimehic et al. (2023) for young adults in Switzerland. Here, users often begin sessions with a specific task in mind (e.g., reading news) but become diverted to other forms of mobile phone use.

At the same time, the association between social media usage and increased news consumption supports findings from Barnidge and Xenos (2024), who identified social media as a trigger for news browsing for young adults and the general population in the United States. In our case, different types of social media were associated with different levels of news use within the same period. While the use of Instagram and X/Twitter was associated with an increase in news use, TikTok stood out with no effect. However, we cannot make any causal claim about this phenomenon; the increased news use may have been instigated by social media posts, but the data are not detailed enough to test the direct causality.

Despite these contributions, our study has limitations. We focused exclusively on smartphone usage, leaving gaps in understanding news consumption through other channels such as broadcast media, television, print, or desktop computers. Notably, Yang et al. (2020) highlighted significant differences between mobile and desktop news consumption, with mobile users encountering a broader variety of news. Additionally, we could not determine the specific content participants consumed on social media or distinguish between news and non-news content on news websites. Future tracking studies should aim to address these gaps. Differentiating between specific types of content consumed on news websites or social media platforms could provide a more nuanced understanding of the interaction between social media use and news engagement, and richer insights into how situational and individual factors shape mobile phone use.

The focus of this study, as part of a larger research project, was specifically on young people, and some of their media habits cannot be generalized to the population as a whole. This may explain why some of our findings deviate from the literature. As an effect of our recruitment strategy, which mainly relied on Instagram ads, our sample was skewed toward better-educated and digitally savvy individuals and females. To account for sampling bias to some extent, we controlled for age and gender. Other demographic variables were considered but excluded for practical reasons. Age posed modeling challenges, as it was closely linked to education and income – older participants were more likely to have completed their degree, hold full-time jobs, and earn higher incomes. While income and education are valid predictors of news use in the general population, young adults are in a transitional life stage where these factors are less stable. Therefore, variables related to income and socioeconomic status were not included in the model. Despite the limitations, we expect some generalizability for well-educated individuals with an affinity for social media.

We must also be careful not to correlate time spent reading news with interest in or engagement with a topic. Groot Kormelink and Costera Meijer (2020) have shown that an individual's interest in a topic might lead to quicker, more efficient scanning for news topics, and that opening a news article does not always mean reading it; time on a news website does not necessarily correlate with information received.

Nevertheless, our findings reveal that news consumption is far from static, varying with time of day, session progression, and social media usage. These insights offer practical implications for researchers studying mobile use habits. As our study finds that news usage differs by time of day, as well as within browsing sessions, researchers should be more wary of responses collected at particular times of day. As temporal and situational factors continue to shape information-seeking behaviors and mobile phones continue to be inexorably tied to our means of gathering information and entertaining ourselves, further exploration of these dynamics will be essential. Future research could produce more detailed accounts of individuals’ habits and pathways to news on mobile devices.

## Supplemental Material

sj-docx-1-mmc-10.1177_20501579251376418 - Supplemental material for Days, Schedules, and Attention: How Time Affects Mobile News Consumption Among Young Swiss People Supplemental material, sj-docx-1-mmc-10.1177_20501579251376418 for Days, Schedules, and Attention: How Time Affects Mobile News Consumption Among Young Swiss People by Morley Weston, Daniel Vogler, Adrian Rauchfleisch, Pascal Jürgens and Mark Eisenegger in Mobile Media & Communication
