# Supplementary material for: Days, Schedules, and Attention: How Time Affects Mobile News Consumption Among Young Swiss People
Source: Mob Media Commun. 2025 Sep 25;14(2):324–45. doi: 10.1177/20501579251376418 (PMC13082691; doi:10.1177/20501579251376418)
Supplement: sj-docx-1-mmc-10.1177_20501579251376418 - Supplemental material for Days, Schedules, and Attention: How Time Affects Mobile News Consumption Among Young Swiss People [file sj-docx-1-mmc-10.1177_20501579251376418.docx]

# 7. Appendix

## Appendix 1: Results tables

| **Parameter** | **Median** | **CI low** | **CI high** | **pd** | **ROPE %** | **ESS** |
| --- | --- | --- | --- | --- | --- | --- |
| Intercept | -7.22 | -7.44 | -7.00 | 100% | 0% | 491.75 |
| Session Progress | -0.35 | -0.39 | -0.31 | 100% | 0% | 18718.48 |
| Time Online Ratio | 3.89 | 3.71 | 4.08 | 100% | 0% | 18450.97 |
| Lagged News | 1.12 | 1.09 | 1.15 | 100% | 0% | 19541.16 |

**Table 2:** Posterior distributions for the base model. The model converged, with all Rhats below 1.01. For all variables, the credible interval was set to 0.95, and region of practical equivalence (ROPE ) set to -0.181 to 0.181.

| **Parameter** | **Median** | **CI low** | **CI high** | **pd** | **ROPE %** | **ESS** |
| --- | --- | --- | --- | --- | --- | --- |
| Intercept | -8.54 | -9.89 | -7.07 | 100% | 0% | 1406.55 |
| Age | 0.09 | 0.02 | 0.15 | 100% | 100% | 1372.80 |
| Gender | 0.35 | 0.06 | 0.63 | 99% | 11% | 1270.47 |
| Time Online Ratio | 2.66 | 2.46 | 2.85 | 100% | 0% | 66264.50 |
| Lagged News | 0.96 | 0.88 | 1.04 | 100% | 0% | 16494.53 |
| Session Progress | -0.29 | -0.34 | -0.24 | 100% | 0% | 62985.88 |
| At Work | -0.08 | -0.14 | -0.02 | 99% | 100% | 42155.60 |
| Is Weekend | -0.10 | -0.14 | -0.06 | 100% | 100% | 51466.24 |
| Is Referendum Day | 0.40 | 0.33 | 0.47 | 100% | 0% | 58929.27 |
| Before 9:00 | -0.12 | -0.20 | -0.05 | 100% | 97% | 35060.27 |
| 09:00 To 11:59 | -0.02 | -0.07 | 0.03 | 74% | 100% | 24243.92 |
| 15:00 To 17:59 | -0.06 | -0.11 | -0.01 | 99% | 100% | 25039.78 |
| 18:00 To 20:59 | -0.03 | -0.08 | 0.02 | 88% | 100% | 25724.25 |
| 21:00 And After | -0.21 | -0.27 | -0.16 | 100% | 8% | 25609.24 |
| Lagged News: Before 9:00 | 0.23 | 0.06 | 0.39 | 100% | 28% | 30262.86 |
| Lagged News: 09:00 To 11:59 | 0.10 | -0.01 | 0.21 | 96% | 96% | 22826.36 |
| Lagged News: 15:00 To 17:59 | 0.00 | -0.11 | 0.11 | 51% | 100% | 21062.42 |
| Lagged News: 18:00 To 20:59 | 0.07 | -0.04 | 0.17 | 89% | 100% | 20996.62 |
| Lagged News: 21:00 And After | 0.17 | 0.05 | 0.28 | 100% | 61% | 22121.23 |
| Visited Twitter | 2.55 | 2.50 | 2.59 | 100% | 0% | 55924.74 |
| Visited Instagram | 0.59 | 0.56 | 0.62 | 100% | 0% | 62744.88 |
| Visited Tiktok | 0.10 | 0.04 | 0.16 | 100% | 100% | 54717.62 |

**Table 3:** Posterior distributions for the full model. The model converged, with all Rhats reaching 1.00. For all variables, the credible interval was set to 0.95, and region of practical equivalence (ROPE ) set to -0.181 to 0.181.

## Appendix II: Model specifications


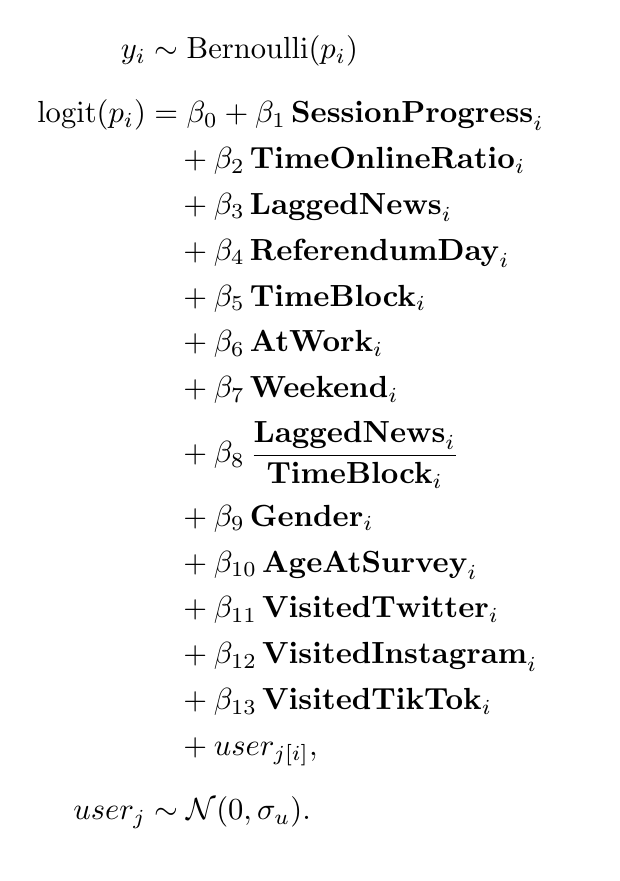


We estimated a multilevel logistic regression model predicting the likelihood of reading news at the observation level, with a random intercept for each user to account for repeated measures. Summary statistics for all variables included can be seen in Table 1. The model was estimated in a Bayesian framework using the brms package with cmdstan as the backend. Priors for all variables were set as Cauchy distributions centered on 0 with a scale parameter of 2.5. The model used 4 chains, each with 10,000 iterations (2,000 warmup), and parallel computation with 4 cores and 2 threads per chain.

## Appendix III: Sensitivity Analysis


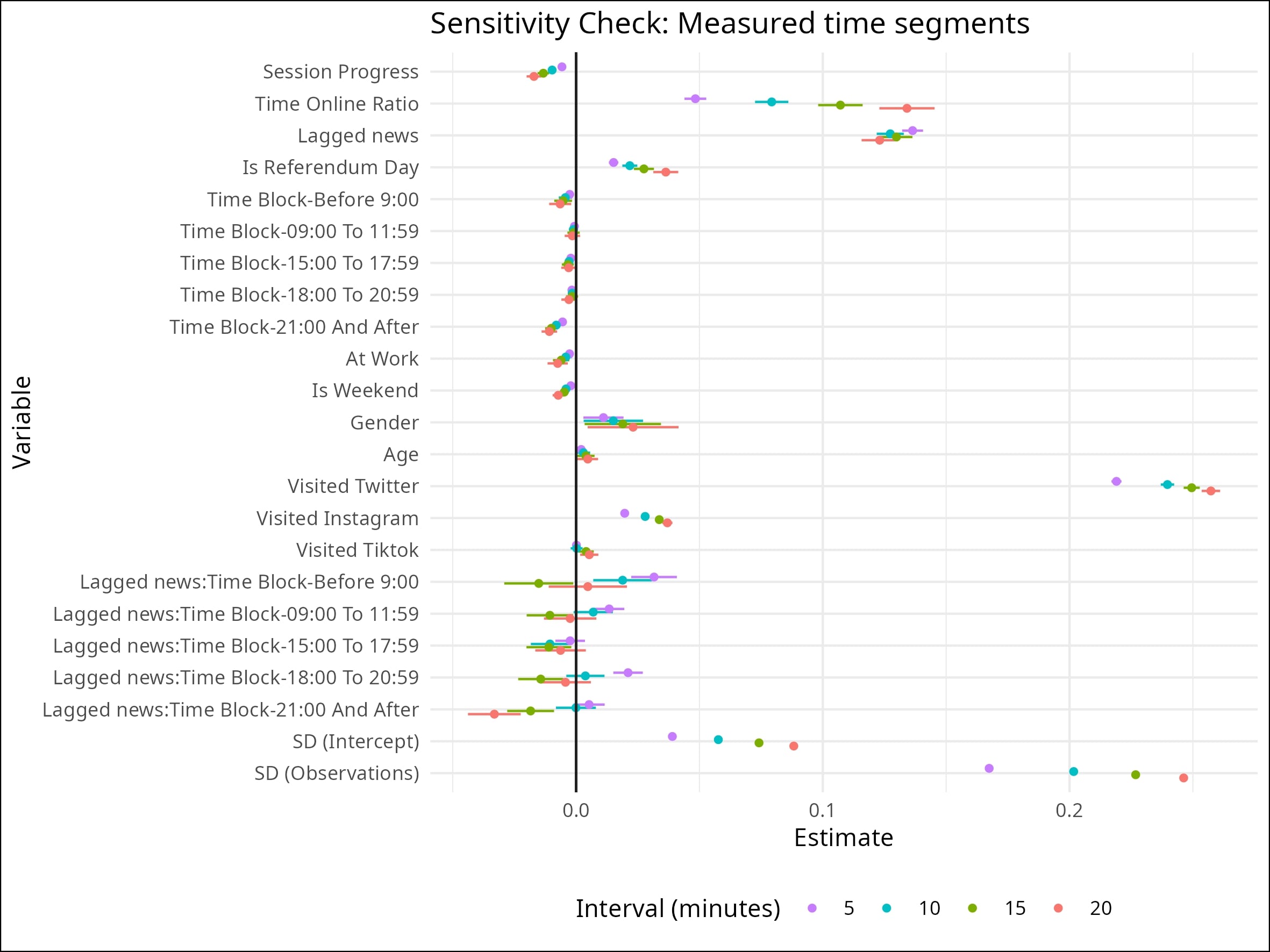


**Figure 4:** Sensitivity analysis on length of time segments measured. Means and 95 percentiles are marked in different segments above.

To ensure that the 10-minute time segments weren’t strongly affecting the results of the analysis, we conducted several variations on the analysis using different time segments. We first conducted the same data preparation using 5-, 10-, 15-, and 20-minute segments. We then fitted a mixed effects frequentist linear model using the same formula as the main model. While some interaction effects differed due to the data preparation, most variables remained consistent with segments of different lengths. Because the reason gathering the data into 10-minute segments was to reduce the total amount of data and ease computational load with Bayesian modeling, a frequentist model was chosen for this check. Thus, the results here are not 100% equivalent to those in the main model, but merely serve as a check to the stability of the data when binned into different lengths of time. Modeling was done with the lme4 package in R.
